# Sequencing and Differential Analysis of miRNA in the Ovaries of Kazakh Horses During Estrus and Anestrus States

**DOI:** 10.3390/ani16142197

**Published:** 2026-07-15

**Authors:** Jiahao Liu, Xinkui Yao, Jun Meng, Jianwen Wang, Yaqi Zeng, Wanlu Ren

**Affiliations:** 1 College of Animal Science, Xinjiang Agricultural University, Urumqi 830052, China; ljh072412@163.com (J.L.); yaoxinkui@xjau.edu.cn (X.Y.); mengjun@xjau.edu.cn (J.M.); dkwjw@xjau.edu.cn (J.W.); zengyaqi@xjau.edu.cn (Y.Z.); 2 Xinjiang Key Laboratory of Equine Breeding and Exercise Physiology, Urumqi 830052, China

**Keywords:** Kazakh horse, ovary, miRNA, oestrous cycle

## Abstract

To reveal the regulatory role of miRNA in the estrous cycle transition of Kazakh horse ovaries and improve their reproductive efficiency, this study took the ovaries of Kazakh horses in the estrus period (DY group) and the quiescent period (DB group) as research objects. Small RNA sequencing and bioinformatics analysis were used to systematically analyze the expression characteristics and functional pathways of miRNA under the two physiological states. A total of 161 million high-quality miRNA tags were obtained through sequencing. Principal component analysis showed that the intra-group repeatability of the two groups of samples was good, and the expression patterns between the groups were significantly different. A total of 259 differentially expressed miRNAs (DEmiRNAs) were identified between the two groups, including eca-miR-21, eca-miR-378, eca-miR-142-3p, eca-miR-196a, and eca-miR-889, among which 99 were significantly up-regulated and 160 were significantly down-regulated. After screening with |log_2_FC| ≥ 1.5, *p* ≤ 0.05 and *q* < 0.05 as strict multiple-testing correction thresholds, a total of 227 valid DEmiRNAs were retained, including 86 upregulated and 141 downregulated miRNAs; the remaining 32 transcripts showed nominal differential expression only without statistical significance after FDR correction. GO functional enrichment indicated that the differentially expressed miRNAs were mainly involved in biological processes such as cellular processes, biological regulation, molecular binding, and catalytic activity. KEGG enrichment showed that they were significantly enriched in pathways closely related to follicular development, ovulation, and luteal function, such as the Ras signaling pathway and actin cytoskeleton regulation pathway. The RT-qPCR verification results were highly consistent with the sequencing data, confirming the reliability of the analysis results. The study demonstrated that the differentially expressed miRNAs could regulate processes such as cell proliferation, apoptosis, steroidogenesis, and follicular development by targeting key signaling pathways, mediating the sequential transition of the estrous–anestrus state of Kazakh horse ovaries. The present study aimed to identify differentially expressed miRNAs in ovarian tissues of Kazakh mares between anestrus (DB) and estrus (DY) states via small RNA sequencing, reveal the miRNA-mediated molecular regulatory mechanisms of seasonal ovarian cycle transition, and provide candidate molecular markers and theoretical basis for genetic improvement of reproductive performance in Kazakh indigenous horses.

## Simple Summary

Kazakh horses are important indigenous equines in China, but their reproductive efficiency is limited by irregular estrous cycles. To reveal the regulatory role of miRNAs in ovarian function, we performed small RNA sequencing on ovaries from estrus and quiescent mares. We obtained 161 million high-quality miRNA tags and identified 259 differentially expressed miRNAs (99 up-regulated, 160 down-regulated). Functional enrichment showed these miRNAs were mainly involved in cellular processes, biological regulation, and pathways critical for folliculogenesis and ovulation, including Ras signaling and actin cytoskeleton regulation. RT-qPCR validated the sequencing data. Our results demonstrate that miRNAs mediate the estrus–quiescence transition by targeting key signaling pathways, providing molecular targets for improving reproductive traits in Kazakh horses. The present study aimed to identify differentially expressed miRNAs in ovarian tissues of Kazakh mares between anestrus (DB) and estrus (DY) states via small RNA sequencing, reveal the miRNA-mediated molecular regulatory mechanisms of seasonal ovarian cycle transition, and provide candidate molecular markers and theoretical basis for genetic improvement of reproductive performance in Kazakh indigenous horses.

## 1. Introduction

The ovary is a core organ of the reproductive system in mammals, and its development status and functional regulation directly determine the estrous cycle, ovulation efficiency and reproductive performance of animals, which is a key link for maintaining the continuation of the population and production efficiency [1,2,3]. The Kazakh horse, as an indigenous horse breed in Xinjiang, China, has unique genetic characteristics such as cold resistance, coarse feed tolerance, strong adaptability, and excellent meat and milk production performance [4,5,6]. It is an important pillar of local animal husbandry and an important part of China’s livestock and poultry genetic resources. Under natural breeding conditions, Kazakh horses generally have problems such as irregular estrous cycles and long estrous quiescence periods, resulting in low reproduction rates, which seriously restrict the large-scale development of the Kazakh horse industry and the efficient utilization of local excellent genetic resources [7]. Therefore, in-depth exploration of the molecular regulatory mechanism of estrous and quiescent states of the ovary in Kazakh horses and the identification of key molecules involved in estrous regulation have important theoretical value for improving their reproductive performance, optimizing breeding strategies, and protecting local excellent genetic resources.

MicroRNAs (miRNAs) are a class of endogenous non-coding single-stranded RNAs with a length of approximately 18–25 nucleotides. They achieve post-transcriptional regulation of target genes by complementary binding to the 3′ untranslated region (3′UTR) of the target gene mRNA, thereby participating in various physiological processes such as cell proliferation, differentiation, apoptosis, and hormone secretion [8,9]. In recent years, the role of miRNAs in the development and estrous cycle regulation of mammalian ovaries has become a critical research area, demonstrating that miRNAs participate in key processes such as follicle development, ovulation, and corpus luteum formation and degeneration through regulating the functions of ovarian granulosa cells and theca cells, and their abnormal expression is closely related to ovarian dysfunction, abnormal estrus, and other reproductive disorders [10]. Currently, research on miRNAs and ovarian development and estrous cycle regulation mainly focuses on human [11], mice [12], cattle [13], and pigs [14] species. Multiple key miRNAs related to follicle development and steroid hormone synthesis have been identified, such as *miR-143*, *miR-181a*, and *miR-21*. These miRNAs participate in ovarian function regulation by regulating their target genes such as PTEN, Bcl-2, and CYP19A1. Liu et al. [15] studied that miRNAs play a crucial role in the follicle development process of pigs. Among them, *ssc-miR-320*, *ssc-miR-423*, and *ssc-miR-451* are rich in processes such as cell proliferation and apoptosis, estrogen signaling pathway, DNA damage, hypoxia and ROS processes, miRNA maturation and function, and ubiquitin processes. These miRNAs and their target genes may play an important role in the regulation of ovarian follicle development.

At present, there are few reports on the differences in miRNA expression between the ovaries of mares in estrus and the quiescent state. Therefore, this study takes the ovarian tissues of mares in estrus and quiescence as the research objects, takes miRNA as the research entry point, combines high-throughput sequencing technology and bioinformatics analysis methods, and systematically conducts the analysis of the expression profile differences of miRNA between the two. Systematically analyze the miRNA differential expression characteristics and regulatory mechanisms of the ovaries of mares in estrus and quiescence, and identify DEmiRNAs and related signaling pathways in the ovarian tissues. The present study aimed to identify differentially expressed miRNAs in ovarian tissues of Kazakh mares between anestrus (DB) and estrus (DY) states via small RNA sequencing, reveal the miRNA-mediated molecular regulatory mechanisms of seasonal ovarian cycle transition, and provide candidate molecular markers and theoretical basis for genetic improvement of reproductive performance in Kazakh indigenous horses.

## 2. Materials and Methods

### 2.1. Experimental Animal

This experiment was conducted in Tacheng Prefecture, Xinjiang in February 2025. The sampling month (February) corresponds to seasonal anestrus for local Kazakh horses due to short photoperiod and low ambient temperature. To obtain actively estrous mares (DY group), two estrus synchronization treatments were implemented: each mare received intramuscular injection of 0.2 mg cloprostenol followed by 1000 IU equine chorionic gonadotropin (eCG) 7 days later. Transrectal ultrasonography was performed daily to monitor ovarian follicular dynamics; individuals with dominant preovulatory follicles (≥35 mm diameter) and typical estrous behavioral signs were classified into the DY estrus group. Mares without dominant follicles and persistent inactive ovarian morphology were assigned to the DB quiescent group. All ovarian tissues were collected within 12 h after ultrasound confirmation. Twelve horses of the same breeding condition were selected as experimental animals and divided into two groups: the estrus period (DY group, n = 6) and the quiescent period (DB group, n = 6). Ovarian tissues were collected by slaughter and quickly placed in liquid nitrogen for storage for future use. All the horses were housed in the same pen in different compartments, and were uniformly fed high-quality dry alfalfa and corn kernels, and provided with sufficient drinking water.

### 2.2. RNA Extraction and Quality Analysis

According to the method provided by the manufacturer, total RNA was extracted using the Trizol reagent kit (Invitrogen, Carlsbad, CA, USA). The quality of the RNA was evaluated using the Agilent 2100 bioanalyzer (Agilent Technologies, Palo Alto, CA, USA) and detected by agarose gel electrophoresis without RNase. Total RNA integrity and purity of ovarian samples were comprehensively assessed via three independent methods: (1) agarose gel electrophoresis to detect intact 28S/18S rRNA bands without obvious degradation; (2) Agilent 2100 Bioanalyzer to calculate RNA integrity number (RIN); only samples with RIN ≥ 7.0 were retained for subsequent library construction; (3) Nanodrop spectrophotometry to detect OD260/280 and OD260/230 values for removing RNA with protein or phenol contamination. Purified ovarian RNA meeting all quality criteria was subjected to miRNA enrichment and library construction. Subsequently, miRNA was purified, fragmented, and reverse transcribed into complementary DNA (cDNA). The reverse transcription of cDNA was performed using the NEBNext Ultra RNA Library Construction Kit (suitable for Illumina, NEB #7530, New England Biolabs, Ipswich, MA, USA). The connection reaction was purified using AMPure XP magnetic beads (New England Biolabs, Ipswich, MA, USA) (1.0X). Subsequently, polymerase chain reaction (PCR) amplification was carried out. The obtained cDNA library was sequenced using the Illumina Novaseq6000 by Gene Denovo Biotechnology Co., Ltd. (Guangzhou, China).

### 2.3. Data Quality Control and Verification

The miRNA library consists of 3 micrograms of RNA from each sample. Twelve libraries for the ovaries of Kazakh horses were prepared using NEBNext^®^ Multiplex miRNA RNA Library Illumina^®^ (NEB, Ipswich, MA, USA) for sequencing. The PCR products were detected and purified on an 8% polyacrylamide gel (100 volts, 80 min). DNA fragments matching 140–160 base pairs were dissolved in 8 microliters of elution buffer. Finally, the library quality was evaluated using the Agilent Bioanalyzer 2100 system equipped with a DNA high-sensitivity chip. The library was sequenced on the Illumina HiSeq 2500 platform (Illumina, San Diego, CA, USA) to generate 50-base-pair single-end reads.

### 2.4. Correlation Analysis Among Samples

Based on the gene expression data, principal component analysis (PCA) was conducted using the R software version 4.3.1. By employing dimension reduction methods, the distance relationships among the samples were analyzed to evaluate the differences in expression patterns between the DB group and the DY group, as well as the consistency within the groups. Correlation analysis was performed to assess the gene expression relationships among the samples, and heatmaps were used to visualize the obtained correlation coefficients to display the pairwise correlations among the samples. The following criteria were used to identify significantly differentially expressed transcripts: |log2 fold change| ≥ 1.5 and *p* ≤ 0.05, while *q* ≤ 1.00 was used to correct the *p* value calculation. The volcano plot and heatmap of differentially expressed miRNAs in the ovary were generated using the DESeq2 R package.

### 2.5. GO and KEGG Enrichment Analysis

Enrichment analysis was conducted for the differential expression of miRNAs, and GO and KEGG pathway analyses were performed for annotation. The KOBAS software (v 3.0.0) was used to test the statistical enrichment of DEGs in KEGG pathways, and the GOseq software (v 1.64.0) was used for GO functional analysis. The significant enrichment criterion was a *p* value less than 0.05.

### 2.6. RT-qPCR Verification

To identify individual miRNAs, total RNA was reverse-transcribed into cDNAs. The primer information for RT-qPCR was used. qRT-qPCR was performed on the CFX Connect fluorescence quantitative PCR instrument (Bio-rad), with each sample analyzed in triplicate. (The primer information is provided in Supplementary Table S1).

## 3. Results and Analysis

### 3.1. RNA-Seq Data Analysis

A total of 12 cDNA libraries were obtained in this study. As shown in Table 1, the miRNA transcriptome of ovarian tissue generated approximately 164 million (average 13,704,928.58) clean reads. After filtering out low-quality reads from the data, 163 million (average 13,656,170.25) reads were obtained. Finally, 161 million (average 13,375,058.33) high-quality miRNA sequencing tags were obtained for subsequent analysis; as shown in Table 2, by comparing the relative abundance values of miRNAs in ovarian tissue, it can be known that the proportions of rRNA, snRNA, snoRNA and tRNA among the total relative abundance values are 0.188%, 0.037%, 1.515% and 0.24% respectively. In total, the available abundance values are approximately 157 million (average 13,085,545.08).

### 3.2. Analysis of Expression Patterns of Ovarian Tissue Samples from Kazakh Horses

**Figure 1:**
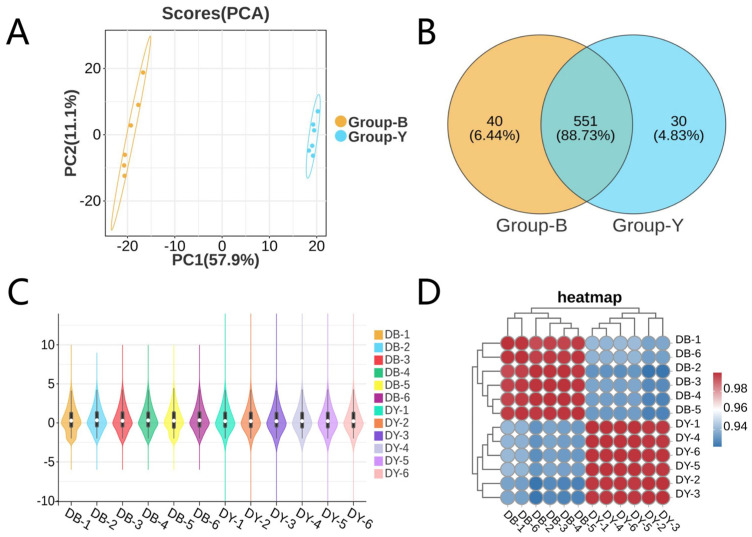
Correlation Analysis Chart Between DB and DY Groups (**A**) PCA Plot of Samples from Group DB and Group DY; (**B**) Venn Diagram for Groups DB and DY; (**C**) Violin Plot of Samples Between Groups DB and DY; (**D**) Correlation Heatmap Between Group DB and Group DY. Abbreviations: DB = quiescent mare group; DY = estrus mare group.

Based on the correlation analysis results between the ovarian tissue samples of sexually active and quiescent states of Kazakh horses, as shown in Figure 1A, in the principal component (PCA) analysis of the DB group and the DY group, PCA1 is the first principal coordinate, representing a contribution rate of 57.9%; PCA2 is the second principal coordinate, representing a contribution rate of 11.1%. The intra-group clustering of the samples in both groups is compact, while the inter-group clustering is loose, indicating significant differences between the two groups. As shown in Figure 1B, the number of miRNAs in the DB group is 591, and that in the DY group is 581, and the total number of miRNAs in both groups is 551. According to the results in Figure 1C,D, due to the relatively small differences in individual expression conditions among different samples, and the overall consistency of expression levels among the samples, the correlation among the samples in each group also shows a similar stable expression trend. (See Supplementary Table S2).

### 3.3. Analysis of Differential Expression Patterns of Ovarian Tissue Samples from Kazakh Mares

**Figure 2:**
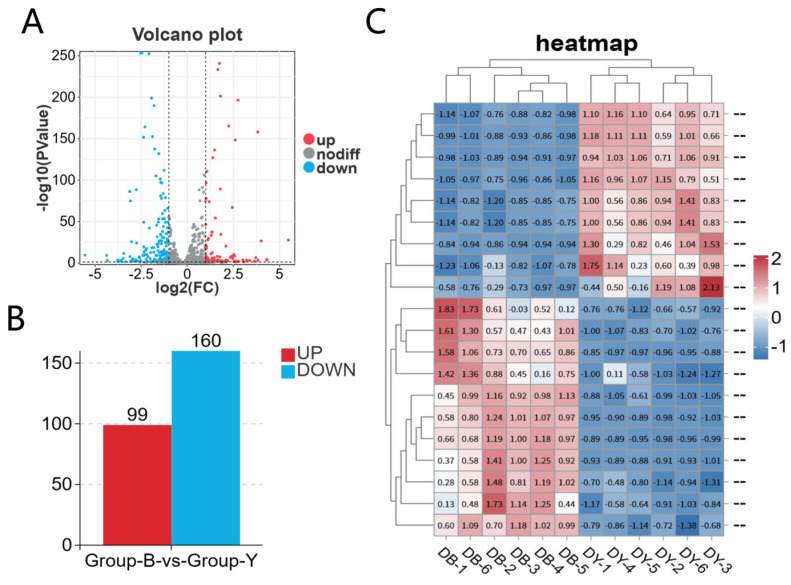
Differential Expression Analysis Diagram for Groups DB and DY. (**A**) Volcano Plot of Differentially Expressed Genes Between Group DB and Group DY; (**B**) Bar Chart of Differentially Expressed Genes Between Group DB and Group DY; (**C**) Heatmap of Differentially Expressed Genes Between Group DB and Group DY. Abbreviations: DB = quiescent mare group; DY = estrus mare group. Note: In (**A**,**B**), ‘up’ and ‘down’ represent upregulated and downregulated miRNA expression in ovarian tissue, respectively. Upregulation: (log_2_FC > 1 & adjusted *p* < 0.05), downregulation: (log_2_FC < −1 & adjusted *p* < 0.05). In (**C**), the horizontal axis represents individual samples, the vertical axis represents expression levels, and the transition from blue to red indicates progressively increasing upregulation.

By analyzing the differentially expressed gene expression results of the ovarian tissues from two groups of Kazakh horses, it can be seen from Figure 2A,B that the DB group and the DY group jointly identified 259 DEmiRNAs including *eca-miR-21*, *eca-miR-378*, *eca-miR-142-3p*, *eca-miR-196a* and *eca-miR-889*. Among them, 99 genes showed upregulated expression and 160 genes showed downregulated expression. After screening with |log_2_FC| ≥ 1.5, *p* ≤ 0.05 and *q* < 0.05 as strict multiple-testing correction thresholds, a total of 227 valid DEmiRNAs were retained, including 86 upregulated and 141 downregulated miRNAs; the remaining 32 transcripts showed nominal differential expression only without statistical significance after FDR correction. The clustering analysis results of the samples are shown in Figure 2C. The ovarian tissue samples show high repeatability among the different groups, revealing significant differences within each group. (See Supplementary Table S3).

### 3.4. GO Functional Annotation and KEGG Enrichment Analysis of miRNAs in Ovarian Tissues of Kazakh Horses

To analyze the functions of DEGs in different ovarian tissue samples, GO and KEGG enrichment analyses were conducted on the DEGs. The functional enrichment analysis of DEGs was divided into three categories: biological process (BP), molecular function (MF), and cellular component (CC).

**Figure 3:**
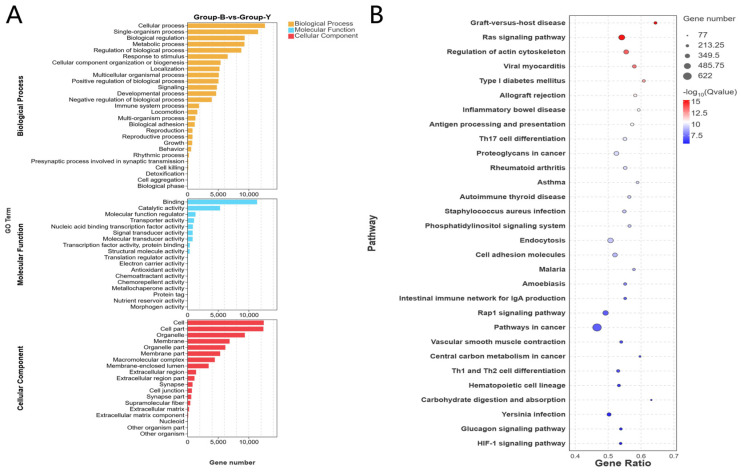
GO and KEGG Enrichment Analysis Diagram for Groups DB and DY. (**A**) GO Annotation Entry Diagram for Group DB and Group DY; (**B**) KEGG Enrichment Analysis Diagram for Groups DB and DY. Abbreviations: DB = quiescent mare group; DY = estrus mare group. Note: In (**A**), the horizontal axis represents each DEG, while the vertical axes denote biological processes (BP), molecular functions (MF), and cellular components (CC). (**B**) depicts the top 20 pathways with the lowest Q-values. The vertical axis displays pathway names, and the horizontal axis shows gene ratios. Count values increase from left to right, with blue to red indicating progressively higher Q-values.

As shown in Figure 3A, the GO annotation results of DB group and DY group indicate that DEmiRNAs are mainly enriched in annotation items such as Cellular process (BP), Single-organism process (BP), Biological regulation (BP), Binding (MF), Catalytic activity (MF), Molecular transducer activity (MF), Cell (CC), Cell part (CC), and Organelle (CC). As shown in Figure 3B, the KEGG enrichment analysis of G1 group and G3 group indicates that DEmiRNAs are mainly enriched in signaling pathways such as Ras signaling pathway, Pathways in cancer, and Regulation of actin cytoskeleton. (See Supplementary Table S4).

### 3.5. miRNA–mRNA Interaction Network

**Figure 4:**
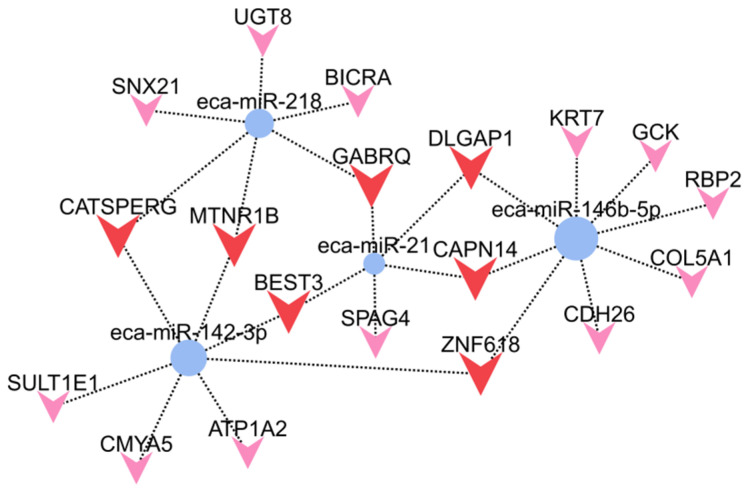
miRNA–mRNA network diagram. Note: miRNA: Blue circle, the size of the node is enlarged according to the degree of connection (*eca-miR-146b-5p* has the largest size) mRNA: Pink triangle, among which the red triangle represents the target genes regulated by multiple miRNAs.

This study dataset includes 4 miRNAs and 19 target genes, forming 4 regulatory modules. *eca-miR-21* is identified as the central regulator in the network, sharing the highest number of common target genes with other miRNAs. *eca-miR-218* is a key regulatory molecule in the network, regulating the largest number of genes. Genes such as *CATSPERG*, *SPAG4*, and *MTNR1B* are highly associated with reproduction, circadian rhythms, and cell movement, suggesting that these four miRNAs may be involved in biological processes including reproductive development, cell migration, and metabolic regulation (Figure 4).

### 3.6. Verification of RT-qPCR Results

**Figure 5:**
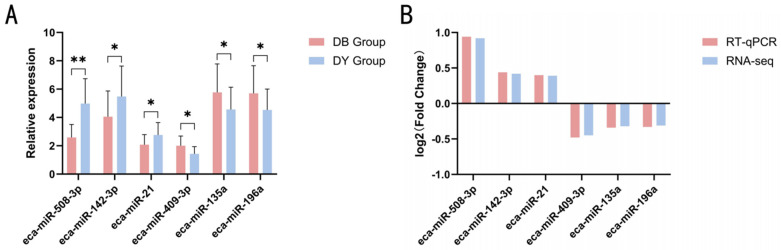
Validation of differentially expressed genes by RT-qPCR. (**A**) Relative expression of differentially expressed genes by RT-qPCR; (**B**) Log2 fold-change comparison between RNA sequencing and RT-qPCR for differentially expressed genes. Abbreviations: DB = quiescent mare group; DY = estrus mare group. Note: The * and ** in the figure indicates a significant difference between the two groups (*p* < 0.05).

To confirm the accuracy of the full transcriptome sequencing data, this study randomly selected *eca-miR-508-3p*, *eca-miR-142-3p*, *eca-miR-21*, *eca-miR-409-3p*, *eca-miR-135a* and *eca-miR-196a* for RT-qPCR verification. The results showed as shown in Figure 5, *eca-miR-142-3p* and *eca-miR-21* were significantly upregulated (*p* < 0.05); eca-miR-508-3p was significantly upregulated (*p* < 0.01); *eca-miR-409-3p*, *eca-miR-135a* and *eca-miR-196a* were significantly downregulated (*p* < 0.05). The expression trends of all genes were consistent in RT-qPCR and RNA-seq results, indicating that the sequenced data and expression status identified in this study are true and reliable, and can be used for subsequent analysis.

## 4. Discussion

The ovary is a complex organ composed of germ cells (referred to as oocytes or eggs) and somatic cells (including granulosa cells, theca cells, and stromal cells), serving as the site for follicle growth and hormone secretion [3,16,17]. The development of the ovary significantly affects the estrus timing of female animals, thereby playing a crucial role in their reproductive performance. Therefore, in this study, by comparing the ovary tissues of estrus and quiescent states of Kazakh horses, 259 differentially expressed miRNAs were screened out. This further confirmed that during the transition from estrus to quiescence, the expression of miRNAs in the ovary tissue underwent specific changes. These differential miRNAs may act as key regulatory factors and participate in the transition of ovary estrus and quiescent states in Kazakh horses.

Through the GO functional annotation and KEGG enrichment analysis of the identified differential miRNAs in ovarian tissues, the results showed that most of the differential miRNAs, such as *eca-miR-142-3p*, *eca-miR-21*, *eca-miR-889*, and *eca-miR-196a*, might be involved in biological processes such as cell proliferation, apoptosis, follicle formation, and follicle degeneration. Therefore, these differential miRNAs play an important role in the transition of the ovary during the estrus and quiescent states. The Ras signaling pathway is the core molecular switch that regulates the periodic function of the ovary. Its activation level strictly changes with the spatiotemporal specificity of the ovary cycle, directly determining the orderly conversion of follicle development, ovulation, and corpus luteum formation and degeneration [18]. The Ras signaling pathway is an important intracellular signal transduction pathway, mainly including core components such as Ras protein, Raf kinase, MEK, and ERK (MAPK). This pathway regulates various biological processes such as cell proliferation, differentiation, survival, and apoptosis, and plays a key regulatory role in the ovary cycle [19,20,21]. Billhaq et al. [22] studied that the regulation of the RAS family is very important in the growth of follicles, ovulation, and the formation and degeneration of the corpus luteum. The RAS family has significant functions in regulating vascular endothelial growth factor (VEGF), insulin-like growth factor (FGF), fibroblast growth factor (IGF), angiopoietin (ANPT), and hypoxia-inducible factor (HIF), indicating that the RAS family is associated with these dominant factors and may participate in the regulation of the corpus luteum of the ovary. Luteinizing hormone (LH) triggers the activation of the Ras/ERK1/2 pathway, which is crucial for ovulation and the terminal differentiation of granulosa cells. LH induces the secretion of EGF-like factors (AREG/EREG) by pre-ovulatory follicles, further activating Ras-ERK through EGFR, becoming the “molecular switch” for ovulation [23]. Studies have shown that the activation of the Ras, MEK1, and ERK1/2 pathways guides the specific cell fate determination of ovarian granulosa cells during growth and pre-ovulatory follicles. In mouse models, the premature expression of mutant stable KRAS (KRASG12D) in granulosa cells completely destroys early follicle development [24]. Chang et al. [25] found that the Ras/MAPK pathway mediates the regulation of neurotrophic factors (NT) and glial cell line-derived neurotrophic factor (GDNF) on ovarian function, including the regulation of ovarian granulosa cells. NTs and GDNF in the ovary play a key role in regulating ovarian function, covering the assembly of ovarian follicles, activation of primordial follicles, growth and development of follicles, steroidogenesis, maturation of oocytes, ovulation, and formation of the corpus luteum. Regulation of actin cytoskeleton is the core regulatory mechanism of ovarian physiological functions and pathological processes, mainly through Rho GTPases (such as RhoA, Cdc42, Rac1) and their downstream ROCK pathway, controlling the polymerization and depolymerization of actin (F-actin), thereby affecting the morphology, migration, contraction, and secretion of ovarian cells. It is associated with the entire process of follicle development, ovulation, hormone synthesis, oocyte maturation, ovulation, and corpus luteum formation, including ovarian function and follicle development, depending on the synergistic and complementary effects between these neurotrophic factors and their corresponding receptors [26,27]. Chen et al. [28] conducted a study and found that Sema4C is a member of the fourth subfamily of semaphorins. It has been proven to play diverse and significant roles in organ development, immune regulation, tumor growth and metastasis. Sema4C is widely expressed in the stroma, follicles and corpus luteum of the mouse ovary. Its expression decreases at specific focal points in the ovaries of middle-aged and older mice. It may regulate the actin cytoskeleton through the RHOA/ROCK1 signaling pathway and play an important role in steroidogenesis of the ovary.

miRNAs exhibit spatiotemporal and cell-specific expression in the animal ovary, serving as core post-transcriptional regulators for follicle development, ovulation, hormone secretion, and follicle closure [29,30,31]. Different species have specific miRNA expression profiles that show temporal and spatial dynamic changes. *miR-21*, *miR-34*, and *miR-145*, among others, show differential expression at different physiological stages. Tscherner’s [32] research findings revealed that miRNAs are highly expressed in oocytes and early embryos, during critical developmental stages with very low or absent transcription. Post-transcriptional gene regulatory mechanisms play a dominant role and have become strong candidate regulatory factors for RNA stability during the transition from the maternal genome to the zygote control during oocyte maturation.

The ovary represents a heterogeneous reproductive organ composed of germ cells and somatic granulosa/theca cells, whose functional state strictly determines mare seasonal cyclicity and fertility. As typical long-day seasonal polyestrous equines, Kazakh horses enter deep winter anestrus (February in Tacheng) under short photoperiod and low ambient temperature: ovarian follicles stagnate below 10 mm, circulating LH and estradiol remain at basal levels, and most growing follicles undergo atresia without dominant follicle deviation or ovulation. During the breeding season with extended daylight, the hypothalamic–pituitary–gonadal (HPG) axis restores GnRH/LH pulsatile secretion, supporting continuous follicular waves, preovulatory follicle maturation and regular ovulation every ~21 days. The present study identified 259 ovarian DEmiRNAs between anestrus (DB) and estrus (DY) Kazakh mares, including core candidates *eca-miR-21*, *eca-miR-142-3p*, *eca-miR-196a* and *eca-miR-889*. This dataset confirms widespread miRNA transcriptional remodeling accompanies the seasonal anestrus-to-estrus transition, and further KEGG enrichment highlighted two core signaling axes—Ras signaling and actin cytoskeleton regulation—as central mediators of equine seasonal ovarian function switching.

Across mammalian models (mice, pigs, cattle), the canonical Ras-Raf-MEK-ERK cascade universally governs granulosa cell proliferation, steroidogenesis and ovulatory luteinization, yet its physiological output differs drastically between seasonal equines and year-round polyestrous livestock [33,34]. Unlike pigs and cattle that maintain stable Ras pathway activity across all seasons, Ras signaling in mares exhibits strict photoperiod-dependent responsiveness to gonadotropin stimulation, which directly explains the long anovulatory window in winter Kazakh mares. During deep anestrus, persistently low pituitary LH secretion fails to trigger granulosa cell EGFR-AREG ligand release, blocking upstream Ras activation; even secondary follicles larger than 20 mm cannot complete dominant follicle selection and regress prematurely [35]. In our estrus DY group, significantly upregulated *eca-miR-21* targets multiple Ras pathway negative regulators, relieving molecular inhibition of ERK1/2 signaling. Activated ERK further transactivates steroidogenic transcription factors *NR5A1* and *LHR1*, driving *CYP19A1*-mediated estrogen synthesis and preovulatory follicle differentiation—processes completely suppressed in winter anestrus ovaries. Previous equine reproductive studies verified that LH surge-induced Ras/ERK activation acts as an obligate “ovulation molecular switch”: ERK cascade knockout abolishes follicle rupture and cumulus–oocyte complex expansion in mares, whereas transitional spring follicles with weak Ras activity produce large anovulatory structures that undergo atresia before the first seasonal ovulation [36]. The downregulated DEmiRNAs (*eca-miR-196a*, *eca-miR-889*) identified herein conversely suppress Ras cascade transduction in anestrus ovaries, locking follicles in growth arrest. While rodent models demonstrate that constitutive Kras mutation disrupts early folliculogenesis, such constitutive activation rarely occurs in native equine ovaries; seasonal Ras suppression driven by miRNA reprogramming represents the primary physiological brake restraining Kazakh horse ovarian activity during cold short-day months.

The activation of primordial follicles is a key step in initiating ovarian function. Studies have shown that the mTORC1 signaling pathway is a key regulatory factor for the activation of pre-granulosa cells, and other pathways such as WNT, NOTCH, KIT, and JNK also participate in the process of primordial follicle activation. miRNAs regulate these signaling pathways to manage the primordial follicle pool [37]. Collectively, this work constructs a seasonal regulatory axis exclusive to indigenous Kazakh horses: short winter photoperiod remodels ovarian miRNA expression profiles to simultaneously repress Ras/MAPK transduction and lock actin cytoskeleton in a static conformation, jointly arresting follicular growth and steroidogenesis to maintain anestrus. With increasing spring daylight, shifts in miRNA abundance (upregulation of *eca-miR-21*, *eca-miR-142-3p*; downregulation of *eca-miR-196a*, *eca-miR-889*) de-repress both signaling cascades, enabling dominant follicle selection, estrogen secretion and eventual ovulation. Most prior ovarian miRNA research focused on non-seasonal mammalian species, lacking context for photoperiod-driven ovarian quiescence; our findings fill this gap by linking equine seasonal reproductive physiology to miRNA-mediated Ras and actin cytoskeleton pathways, offering candidate molecular markers to alleviate irregular estrus and shorten anestrus duration in Xinjiang local horse breeds.

## 5. Conclusions

This study conducted miRNA sequencing analysis on the ovarian tissues of mares during the estrus and quiescent periods. The results showed that DEmiRNAs such as *eca-miR-21*, *eca-miR-378*, *eca-miR-142-3p*, *eca-miR-196a* and *eca-miR-889* were significantly enriched in signaling pathways such as Ras signaling pathway, pathways in cancer and Regulation of actin cytoskeleton. They also played roles in biological processes such as ovarian steroidogenesis, follicle formation and cell cycle regulation, thereby regulating the temporal process of the mare’s ovary transitioning from the quiescent period to the estrus period, and thereby improving the reproductive efficiency of Kazakh mares.

## Figures and Tables

**Table 1:** Overall detection of miRNA sequencing data.

Samples	Clean_Reads	High_Quality	Clean_Tags
DB-1	10,927,999	10,906,351 (99.8019%)	10,713,966 (98.0414%)
DB-2	13,585,535	13,551,386 (99.7486%)	13,345,741 (98.2349%)
DB-3	13,805,300	13,771,164 (99.7527%)	13,581,229 (98.3769%)
DB-4	13,503,155	13,463,898 (99.7093%)	13,248,341 (98.1129%)
DB-5	10,057,193	10,037,064 (99.7999%)	9,881,000 (98.2481%)
DB-6	14,331,888	14,289,038 (99.7010%)	14,033,486 (97.9179%)
DY-1	16,038,620	15,942,338 (99.3997%)	15,612,592 (97.3437%)
DY-2	16,769,741	16,668,943 (99.3989%)	16,121,103 (96.1321%)
DY-3	14,472,244	14,420,179 (99.6402%)	13,985,993 (96.6401%)
DY-4	14,047,914	13,998,964 (99.6515%)	13,718,483 (97.6549%)
DY-5	13,970,479	13,921,526 (99.6496%)	13,625,179 (97.5284%)
DY-6	12,949,075	12,903,192 (99.6457%)	12,633,587 (97.5636%)

Note: Samples: Sample name; clean_reads: Filter the data; high_quality: High-quality readings; clean_tags: High-quality sequencing tags. Abbreviations: DB = quiescent/anestrus group; DY = estrus group.

**Table 2:** miRNA relative abundance value detection data.

Total_Abundance	rRNA_Abundance	snRNA_Abundance	snoRNA_Abundance	tRNA_Abundance	Other_Abundance
10,713,966	16,853 (0.16%)	4115 (0.04%)	126,477 (1.18%)	27,194 (0.25%)	10,539,327 (98.37%)
13,345,741	28,074 (0.21%)	7182 (0.05%)	307,694 (2.31%)	86,392 (0.65%)	12,916,399 (96.78%)
13,581,229	25,444 (0.19%)	6299 (0.05%)	190,855 (1.41%)	53,886 (0.40%)	13,304,745 (97.96%)
13,248,341	26,332 (0.20%)	6622 (0.05%)	204,212 (1.54%)	59,452 (0.45%)	12,951,723 (97.76%)
9,881,000	18,052 (0.18%)	4410 (0.04%)	129,323 (1.31%)	37,710 (0.38%)	9,691,505 (98.08%)
14,033,486	24,018 (0.17%)	5618 (0.04%)	147,817 (1.05%)	38,351 (0.27%)	13,817,682 (98.46%)
15,612,592	32,910 (0.21%)	4508 (0.03%)	367,291 (2.35%)	14,598 (0.09%)	15,193,285 (97.31%)
16,121,103	29,712 (0.18%)	3879 (0.02%)	250,501 (1.55%)	11,872 (0.07%)	15,825,139 (98.16%)
13,985,993	25,427 (0.18%)	3520 (0.03%)	213,687 (1.53%)	9529 (0.07%)	13,733,830 (98.20%)
13,718,483	27,434 (0.20%)	3940 (0.03%)	325,595 (2.37%)	12,997 (0.09%)	13,348,517 (97.30%)
13,625,179	26,582 (0.20%)	3486 (0.03%)	265,808 (1.95%)	10,408 (0.08%)	13,318,895 (97.75%)
12,633,587	23,113 (0.18%)	3173 (0.03%)	213,150 (1.69%)	8657 (0.07%)	12,385,494 (98.04%)

Note: total_abundance: Relative abundance value; rRNA_abundance, snRNA_abundance, snoRNA_abundance, tRNA_abundance: Relative abundance of non-target miRNA; other_abundance: Residual abundance value.

## Data Availability

The data presented in this study are openly available in BioProject (reference number: PRJNA1467629).

## Supplementary Materials

The following supporting information can be downloaded at: https://www.mdpi.com/article/10.3390/ani16142197/s1, Table S1: Primer information; Table S2: miRNA expression level; Table S3: All differentially expressed miRNAs; Table S4: GO and KEGG enrichment analysis.
